# Primary Stability of Implants Inserted into Polyurethane Blocks: Micro-CT and Analysis In Vitro

**DOI:** 10.3390/bioengineering11040383

**Published:** 2024-04-15

**Authors:** Chadi Dura Haddad, Ludovica Andreatti, Igor Zelezetsky, Davide Porrelli, Gianluca Turco, Lorenzo Bevilacqua, Michele Maglione

**Affiliations:** 1 Department of Medicine, Surgery and Health Sciences, University of Trieste, Piazza dell’Ospitale 1, 34129 Trieste, Italy; sdurahaddad@gmail.com (C.D.H.); ludovica.andreatti@gmail.com (L.A.); igor.zelezetsky@units.it (I.Z.); gturco@units.it (G.T.); l.bevilacqua@fmc.units.it (L.B.); 2 Department of Life Sciences, University of Trieste, Via Alexander Fleming 31-B, 34127 Trieste, Italy; dporrelli@units.it

**Keywords:** primary stability, implant site preparation, polyurethane blocks, piezo surgery, magnetic mallet, traditional drills, Osstell, Micro-CT

## Abstract

The approach employed for the site preparation of the dental implant is a variable factor that affects the implant’s primary stability and its ability to integrate with the surrounding bone. The main objective of this in vitro study is to evaluate the influence of different techniques used to prepare the implant site on the primary stability of the implant in two different densities of artificial bone. Materials and Methods: A total of 150 implant sites were prepared in rigid polyurethane blocks to simulate two distinct bone densities of 15 pounds per cubic foot (PCF) and 30 PCF, with a 1-mm-thick simulated cortex. The implant sites were equally distributed among piezoelectric surgery (PES), traditional drills (TD), and black ruby magnetic mallet inserts (MM). Two methods have been employed to evaluate the implant’s primary stability, Osstell and micro-tomography. Results: In the present study, we observed significant variations in the implant stability quotient (ISQ) values. More precisely, our findings indicate that the ISQ values were generally higher for 30 PCF compared to 15 PCF. In terms of the preparation technique, PES exhibited the greatest ISQ values, followed by MM, and finally TD. These findings corresponded for both bone densities of 30 PCF (PES 75.6 ± 1.73, MM 69.8 ± 1.91, and TD 65.8 ± 1.91) and 15 PCF (PES 72.3 ± 1.63, MM 62.4 ± 1.77, and TD 60.6 ± 1.81). By utilizing Micro-CT scans, we were able to determine the ratio of the implant occupation to the preparation site. Furthermore, we could calculate the maximum distance between the implant and the wall of the preparation site. The findings demonstrated that PES had a higher ratio of implant to preparation site occupation, followed by TD, and then the MM, at a bone density of 30 PCF (PES 96 ± 1.95, TD 94 ± 1.88, and MM 90.3 ± 2.11). Nevertheless, there were no statistically significant differences in the occupation ratio among these three approaches in the bone density of 15 PCF (PES 89.6 ± 1.22, TD 90 ± 1.31, and MM 88.4 ± 1.17). Regarding the maximum gap between the implant and the site preparation, the smallest gaps were seen when TD were used, followed by MM, and finally by PES, either in a bone density 15 PCF (PES 318 ± 21, TD 238 ± 17, and MM 301 ± 20 μm) or in a bone density 30 PCF (PES 299 ± 20, TD 221 ± 16, and MM 281 ± 19 μm). A statistical analysis using ANOVA revealed these differences to be significant, with *p*-values of < 0.05. Conclusion: The outcomes of this study indicate that employing the PES technique and osteo-densification with MM during implant insertion may enhance the primary stability and increase the possibility of early implant loading.

## 1. Introduction

Since their invention by Branemark in the late 1970s, dental implants have become the most prevalent method for replacing missing teeth because of their numerous benefits, including the preservation of adjacent structures, enhanced patient comfort and aesthetics, superior function, and longevity [1]. Several factors influence the duration of hard tissue recovery after dental implant insertion. These variables include the macro- and micro-geometry of the implant, its primary stability, the bone’s structure, and the type of occlusion [2].

The most prevalent method for preparing the implant site involves the use of rotary instruments, which comprise a series of calibrated drills supplied by the implant’s manufacturer that are compatible with the implant’s geometry.

While the conventional procedure involving implant TD is indeed reliable, efficient, and standardized, it does have some disadvantages. In fact, the cutting part of the drills is not selective, and this does not prevent injury to delicate anatomical structures like blood vessels and nerves. In addition, the slow rotation speed of the drills may transmit vibrations to the handpiece, which can compromise control during the osteotomy phase. Drilling procedures can lead to mechanical bone trauma and heat-induced bone necrosis, which greatly increases the likelihood of implant osseointegration failure [3].

PES is a surgical technique that emerged in the 1990s. This method utilizes the well-established physical principle of cavitation, which states that ultrasonic micro-vibrations with a modulated amplitude of between 60 and 200 microns can create incisions in even the most densely mineralized tissues, such as bone tissue, enamel, and dentin. The incisions produced by the PES possess several major advantages, including simplicity and efficiency of execution, reproducibility, a standardizable procedure, high precision in creating linear and conservative engravings, minimal soft tissue trauma in the surrounding area, and a significant reduction in harmful complications that may affect the noble anatomical structures in the orofacial region (such as the Schneiderian membrane, inferior alveolar nerve, and arteries) in the case of accidental direct contact [4,5].

The main difference between these first two techniques lies in the fact that the TD produces a specific shape that replicates the macro-geometry of the used implant, whereas the ultrasonic inserts used for site preparation are not implant-specific and can be utilized to insert screws with various morphologies [6].

MM is an innovative instrument that is capable of generating predetermined and adjustable forces at the most suitable moments for application. These forces, of high intensity and short duration, are transmitted to the tip of the osteotome in order to achieve plastic deformation of the bone. The apparatus consists of a handpiece energized with an electronic power supply that regulates the application forces and durations. Different inserts could be attached to the handpiece, which pushes a shock wave on its tip according to the surgical procedures. The following four force modes are available: 75, 90, 130, and 260 daN. The time of impact is 80 μs [7]. The primary advantage of the MM is that the velocity is increased, due to the application of more forces using dynamic-magnetic propulsion, as compared to manual instruments. The forces are concentrated on the treatment area to minimize the scattering of craniofacial mass, leading to enhanced precision. Additionally, the instruments move longitudinally without the risk of deviations caused by varying bone densities, which improves the directionality; moreover, the mechanical frictions that occur are insufficient to raise the temperature of the bone [8].

Osseointegration is defined as the direct structural and functional connection between the bone and the surface of a functionally loaded implant, and it is known that primary implant stability (lack of mobility) during the bone healing period is necessary for osseointegration to occur [9]. Several non-invasive diagnostic devices based on modal analysis (vibration analysis) are available to clinically monitor the implant stability during the healing period, such as Periotest^®^ (Medizintechnik Gulden e.k., Modautal, Germany), dental movement checker, and Osstell^®^ (Osstell AB, Gothenburg, Sweden). Periotest^®^ has been criticized for having insufficient sensitivity to measure implant mobility [10,11]. In addition, dental movement checker, by applying a modest force with a hammer, can compromise the process of osseointegration in a recently placed implant [12].

The primary stability of a dental implant refers to the lack of micro movements exceeding 150 μm after it is inserted. This characteristic is crucial for the successful integration of the implant with the surrounding bone [13,14]. In a clinical setting, the primary stability is mainly estimated by using the following two methods: measuring the peak of the insertion torque (IT) [15,16], which refers to the maximum force used by the implant micromotor to position the implant, or by performing resonance frequency analysis utilizing the Osstell^®^ instrument [17]. Additional indicators of primary stability include the removal torque (RT) [18], which refers to the force required to remove the implant, and the histological evaluation of the contact area between the bone and the implant (bone–implant contact, BIC) [19]. However, the last two measurements are not suitable for clinical use [20].

Osstell^®^ is a technology that measures the primary stability of an implant based on bone quality. It is non-invasive, provides quick results, and is easy to use. Additionally, it allows for a subsequent assessment of osseointegration without causing scars or exposing the area to the risk of infection [17]. Osstell^®^ utilizes resonance frequency analysis (RFA) to quantify stability on an ISQ scale that ranges from 0 to 100 KHz. According to this scale, ISQ values of below 60 indicate low stability, values between 60 and 65 indicate low medium stability, values between 65 and 70 indicate high medium stability, and values equal to or greater than 70 indicate high stability [21]. 

The invention of computed tomography X-ray in the early 1970s revolutionized medical practice. It has become possible to obtain rotated projections from multiple viewing directions in order to reconstruct three-dimensional images. Micro-CT enables the analysis of many samples, including mineralized tissues like teeth and bones, as well as materials such as ceramics, polymers, scaffold biomaterials, and others [22]. Thanks to the non-destructive nature of the imaging technique, it is possible to evaluate the internal properties of the same sample numerous times, and the samples remain available for additional biological and mechanical testing after scanning [23].

Titanium, a highly popular material used for dental implants, exhibits a greater capacity to absorb X-rays compared to bone. This often causes beam-hardening effects, which frequently arise when a polychromatic X-ray beam traverses specific substances [24]. The phenomenon of beam hardening is directly correlated with the thickness of the material through which the beam passes. Therefore, the radiation that passes through the center of the target undergoes a higher level of hardening in comparison to the radiation that travels via the borders. Consequently, the final image will be darker in the center and lighter at the borders. The correction can be achieved by placing a metal plate, such as aluminum, between the beam and the target. This eliminates the beam’s less energetic component before it reaches the target. However, this trick does not entirely resolve the issue, which can be further resolved during the reconstruction process [25]. 

Rigid polyurethane foam represents a valuable resource for biomedical applications, including testing dental implants [26]. This synthetic substance has exhibited uniform cortical bone density and depth and has remained unaffected by desiccation, requiring no exceptional management or conservation [27]. The utilized material does not replicate the structure of human bone; however, it does mimic its mechanical characteristics and has demonstrated a strain–stress curve closely resembling that of human bone, according to ASTM-F-1889-08 standards [28,29].

The originality of this study lies in its comprehensive analysis of implant stability using a variety of preparation techniques (PES, TD, and MM) in two distinct densities of synthetic bone, which mimic the trabecular bone of the maxillary and mandibular jaws. Unlike prior research, which often focuses on a single method or bone density, our study provides a comparative evaluation across multiple techniques, offering a broader understanding of the factors influencing implant stability. Additionally, the utilization of both Micro-CT scans and ISQ measurements provides a unique dual approach to assessing primary stability.

The null hypothesis of our research is that there is no significant difference in the primary stability of dental implants when comparing various site preparation techniques (PES, TD, and MM) in different densities of synthetic bone. Our study seeks to either accept or reject this hypothesis based on the empirical evidence gathered through rigorous testing.

## 2. Material and Methods

Two synthetic bone models made of rigid polyurethane foam blocks (Laminated foam blocks, Sawbones Europe, AB, Malmo, Sweden) were chosen to mimic the trabecular bone of the maxillary and mandibular jaws with the following densities: 15 PCF, 0.24 g/cm^3^, modello 1522-02 Sawbones, and 30 PCF, 0.48 g/cm^3^, modello 1522-04 Sawbones [30]. The trabecular bone blocks were then attached to a 1-mm-thick synthetic cortical bone shell, modello 1522-103 Sawbones, on one side only, simulating the cortical bone layer above the trabecular bone in both models. The polyurethane blocks used for this experiment were all rectangular, with dimensions of 12 × 17 × 4.1 cm.

The implants were inserted with 3 different techniques by a single operator, as follows:

- TD: The drill preparation of the implant was performed with Winsix, Biosafin company (Ancona, Italy). The sequence began with a 2.0-mm-diameter precision pilot drill, followed by conical drills of progressively larger diameters (2.6 mm and 3.0 mm), moreover; the final cutter used for all of the blocks had a diameter of 3.4 mm. The sequence of drills was performed using an implant motor set to a speed of 800 revolutions per minute (rpm) and with the assistance of external cooling. The implants were mechanically screwed at the standard rate of 35 rpm.
- PES: The piezoelectric preparation of the implant site was performed with the S.U.S. (surgery ultrasonic site, Esacrom, Imola, Italy). All S.U.S. have a uniform octagonal star section but vary in diameters and tapers. The sequence comprised 4 successive steps. It began with the insertion of a first guide (ES052XGT), followed by a series of conical inserts with gradually larger diameters of 2.8 (ES02.8T), 3.2 (ES03.2T), and 3.6 mm (ES03.6T), and the frequency of 22-35 kHz. According to the manufacturer’s recommendations, the inserts were used with abundant irrigation, and the operator performed a combination of vertical inward and outward motions together with rotational movements.
- MM: Due to the geometry of the tips, which are conical at the end and then parallel with a diamond-like carbon (DLC) coating, they are able to penetrate bone much more easily than conventional osteotomes [31]. DLC is an innovative carbon-based coating that reduces abrasion, slippage, and chemical-aggression-related issues. This material is distinguished by its high hardness, resistance to wear and attrition, low coefficient of friction, resistance to scratching, and biocompatibility. The inserts had the sequences of 2.26 (BLK-R1), 2.60 (BLK-R2), 3.10 (BLK-R3), and 3.6 mm (BLK-R4).

**Figure 1:**
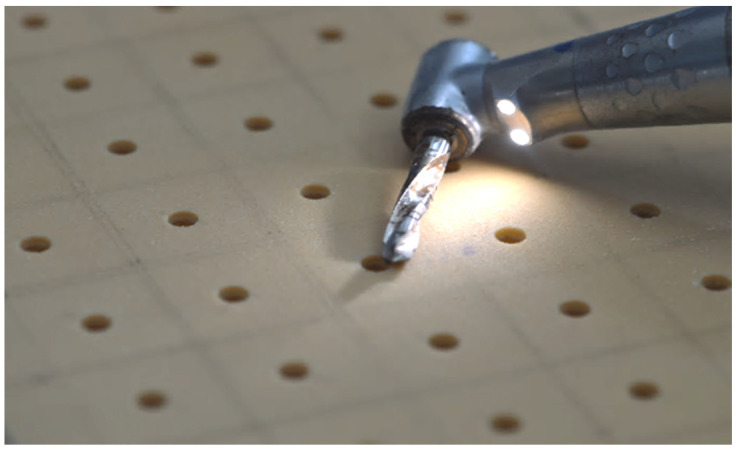
Implant preparation in a grid of 1.5-cm-by-1.5-cm squares on the polyurethane blocks.

A total of 150 implants (Winsix, Biosafin, Ancona, Italy), each measuring 3.8 × 11 mm, were used. These implants had a cylindrical shape with a conical apex and a double-threaded system. The groups were classified based on the bone density and the technique employed for the preparation of the dental implant site. According to the bone density, there were two distinct categories, as follows: one with a bone density of 30 PCF, which mimics the density of the mandibular bone, and another with a bone density of 15 PCF, which closely mimics the density of the maxillary bone. In terms of preparation techniques, we employed the following three different approaches for implant preparation: for group one, we utilized the TD; for group two, we employed the PES approach; and for group three, we used the MM technique. Consequently, there existed a total of six distinct groups. The implants were placed at a constant distance, forming a grid of 1.5-cm-by-1.5-cm squares on the polyurethane blocks (Figure 1). This ensured that the distance between each implant exceeded the minimum distance recommended by clinical guidelines. On the examined polyurethane blocks, 150 implant preparations were conducted, with 25 implants for each group. 

### 2.1. Osstell^®^ IDx Analysis

The Osstell^®^ IDx third generation instrument was utilized to perform the ISQ measurements, and a small aluminum rod, called a SmartPeg, was placed in the implant. The RFA device prompted vibration in the rod by initiating magnetic pulses of varying frequencies. Consequently, the device detected the resonance frequency of the rod while attached to the implant [32]. In order to standardize the procedure, all ISQ measurements were taken with the Osstell handpiece perpendicular to the implant. Three measurements were obtained for each implant, and, subsequently, the mean and standard deviation (SD) were calculated.

### 2.2. X-ray Microcomputed Tomography Analysis

X-ray microcomputed tomography of scaffolds was performed using a custom-made cone beam system called TOMOLAB (Elettra, Trieste, Italy). The samples were positioned on the rotating stage of the device and acquisitions were carried out using the following parameters: source–detector distance (D_SD_): 250 mm, source–object distance (D_SO_): 100 mm, magnification factor (M): 2.5×, binning: 2 × 2, resolution of tomography: 10 µm, tomography dimensions (pixel): 2004 × 1335, slice dimensions (pixel): 1984 × 1984, number of tomography: 1440, number of slices: 1300, electric potential or voltage (E): 200 kV, electric current (I): 200 µA, and exposure time: 1.5 s (without using filters) and 4 s (with aluminum filter). The process of slice reconstruction and the correction of the beam hardening and ring artifacts were performed using the commercial software (Cobra Exxim, Version 5). Input projections and output slices were represented by files (one file per projection and one file per slice) using arrays of 16-bit integers. The software Amira (Version 6.1.1, Thermo Fisher Scientific, Waltham, MA, USA) was used for the co-registration of the volumes of the samples before and after the treatment, to overlap and compare them, and, subsequently, to create the 3D models. The segmentation and the analysis of the samples were performed with the aid of the BoneJ plugin implemented with Fiji software (Version 2.13.0).

One potential solution for handling the issues arising from beam hardening and metal artifacts was utilizing 3D-printed screws fabricated from Nylon PA 12 carbon-filled material. These screws possess an identical morphology and design to those of titanium implants. Therefore, we utilized a protocol consisting of the following: Initially, we performed a cross-sectional CT scan of our implant sites that were prepared using different techniques (TD-PES-MM) on two varying densities of synthetic bone (15 PCF–30 PCF), First Scan (PRE). Then, the 3D-printed screws were inserted manually into the preparation site with a torque that did not surpass 35 Ncm. Next, we completed a second CT scan on the samples after the screw had been inserted, referred to as the Second Scan (POST). Therefore, we used specialist software (in particular, Cobra Exxim) to analyze the data and reconstruct the samples. Due to the fact that each sample was divided into around 1300 slices, with each slice having a thickness of 10 microns, during the CT scan process, the area of the site preparation generated using different approaches was measured on each slice of the PRE scan before inserting the 3D-printed screw. Similarly, the area of the implanted screw was measured on each slice of the POST scan. Then, the cumulative areas of each slice were used to calculate the total volume of the preparation site in the PRE scan and the volume of the printed screw in the POST scan. Finally, to calculate the screw occupation ratio in the preparation site, we divided the entire screw volume by the overall volume of the preparation site. A higher ratio of screws to preparation sites leads to increased primary stability and a higher rate of success [33].

**Figure 2:**
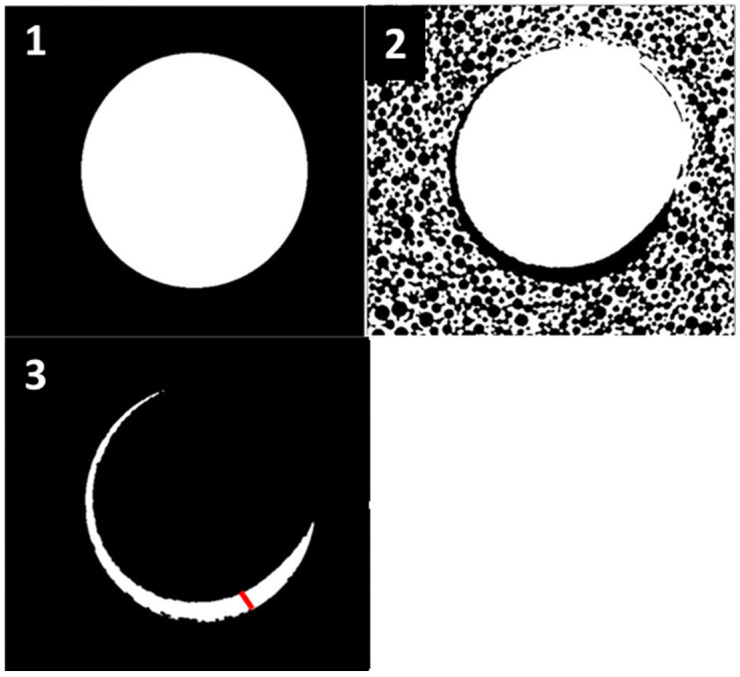
(**1**)—First Scan of the preparation site (pre), (**2**)—Second Scan of the inserted screw (post), (**3**)—overlapping of both scans (pre and post) and determination of the maximum distance between the screw and the bone (red line).

In order to determine the maximum micro-gap between the implant and the preparation site, we used the following protocol: We began by using a specialized software (in this case, Amira by Thermo Fisher Scientific) to adjust the alignment of the scans. This involved aligning the pre-scan, which includes site preparation, and the post-scan, which incorporates a 3D-printed screw, to ensure that both scans have identical rotation and angulation. Subsequently, we measured the difference between the total area of the screw and the total area of the site preparation for each section of our scan. This calculation provided us with a precise estimate of the surface area of the space between the screw and the artificial bone. Furthermore, we could calculate the diameter of this area by employing a particular software (BoneJ plugin, version 7.0.18, in this case). Next, we employed the same approach for every section of each scan to determine the maximum distance between the screw and the synthetic bone in the entire preparation site (Figure 2). The relationship between the primary stability and micro-gap area is inversely proportional. This means that, as the micro-gap area increases, there is an increase in micro-movements. Consequently, a bigger micro-gap has a negative impact on the success of dental implants. This analysis has a very high clinical value, as it directly influences the primary stability of implants and the possibility of immediate loading.

## 3. Results

Osstell^®^ ISQ values: When comparing the bone densities of 30 PCF and 15 PCF together, substantial variations in the ISQ values were observed, with 30 PCF exhibiting superior average results. However, when each bone density was evaluated individually, significant variability in the ISQ values was seen among the three distinct preparation approaches. Within the bone density of 15 PCF, the PES preparations yielded significantly higher ISQ values of 72.3 ± 1.63, compared to the other procedures, indicating high stability on the ISQ scale. There were no statistically significant differences in the ISQ outcomes between the TD and MM methods. The MM demonstrated a slightly higher primary stability than the TD, with ISQ values of 62.4 ± 1.77 and 60.6 ± 1.81, respectively, indicating a medium stability on the ISQ scale. Among the preparation techniques that were analyzed in the bone density of 30 PCF, the following outcomes were observed: The PES demonstrated higher ISQ values of 75.6 ± 1.73 compared to the other preparation techniques, indicating a high stability on the ISQ scale. The comparison of the TD to the MM revealed moderate variations in ISQ values, in which the MM had higher values, with findings of 69.8 ± 1.91 compared to TD’s 65.8 ± 1.5, indicating a medium stability on the ISQ scale for both techniques. A statistical analysis using ANOVA revealed these differences to be significant, with p-values indicating that the PES outperformed the TD and MM (for 30 PCF, PES vs. TD: *p* = 0.032, PES vs. MM: *p* = 0.045; for 15 PCF, PES vs. TD: *p* = 0.038, PES vs. MM: *p* = 0.049), suggesting that the preparation technique influences the implant stability. The complete results are listed in Table 1.

Micro-CT results: In every group, we obtained three Micro-CT scans. Each scan consisted of 1300 slices. We evaluated the 1000 slices located at the central region of the scan, due to the substandard quality of the slices at the periphery. The findings presented in the current study represent the mean value.

- Screw to site preparation occupation ratio: Table 2 demonstrates the occupation rate for all six protocols. Evidently, there were notable variations between the 30 PCF and the 15 PCF bone density measurements, with 30 PCF exhibiting superior average outcomes.

After the individual analysis of each bone density, it was observed that there were no significant differences among the various techniques employed to prepare the implant sites at a density of 15 PCF, and all of the results exhibited a high degree of similarity. At a density of 30 PCF, there were no statistically significant differences identified between the PES and TD techniques. However, on average, the PES had a slightly higher occupation ratio. Nevertheless, both prior techniques yielded a greater ratio of screws occupying the prepared sites compared to MM. The differences among the mean results of our various site preparation approaches are presented in Table 2.

- Screw site preparation micro-gap: The results demonstrated that there were no significant differences in the maximum distance between the 3D-printed screw and the wall of the preparation site when comparing the same preparation techniques in different bone densities, specifically 15 and 30 PCF. Nevertheless, notable disparities emerged when comparing the outcomes of the various approaches. This suggests that the technique employed for the preparation has an important impact on the micro-gap and, consequently, the primary stability of dental implants. The preparations undertook with a bone density of 15 PCF had marginally greater mean values compared to those of 30 PCF. The TD technique demonstrated the smallest micro-gap compared to the other two procedures. When comparing the PES and MM, there were moderate variations observed between these two distinct bone preparation techniques, wherein the PES produced higher outcomes, regardless of whether the bone density was 15 or 30 PCF.

The biggest micro-gap between the screw and bone was seen while employing the PES. The observed effect may be related to the rotational movement (clockwise and counterclockwise) during the vertical insertion and removal of the ultrasonic tips, as instructed by the manufacturer of the S.U.S. (surgery ultrasonic site) (Esacrom, Imola, Italy). The complete results are listed in Table 2.

**Figure 3:**
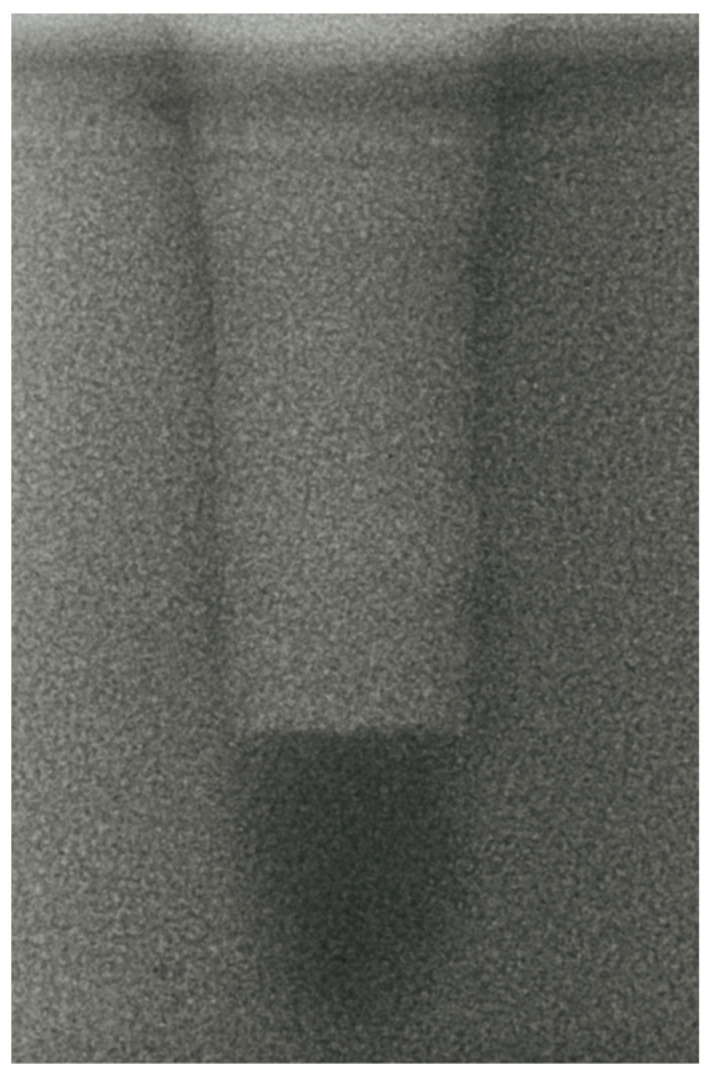
The MM technique (30 PCF).

**Figure 4:**
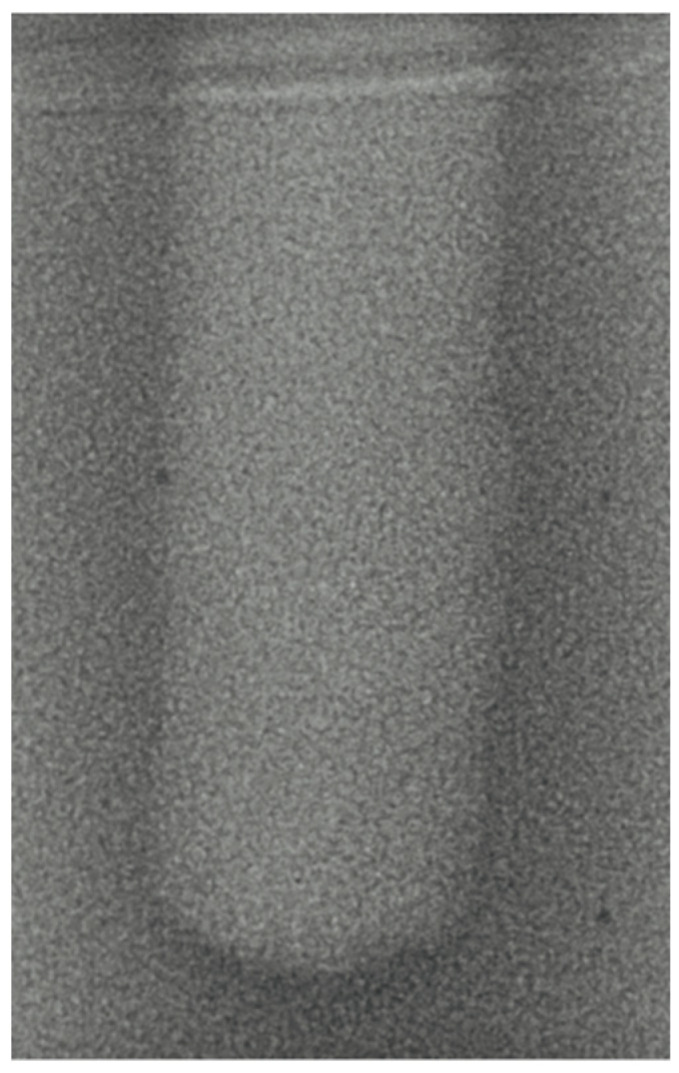
The PES technique (30 PCF).

**Figure 5:**
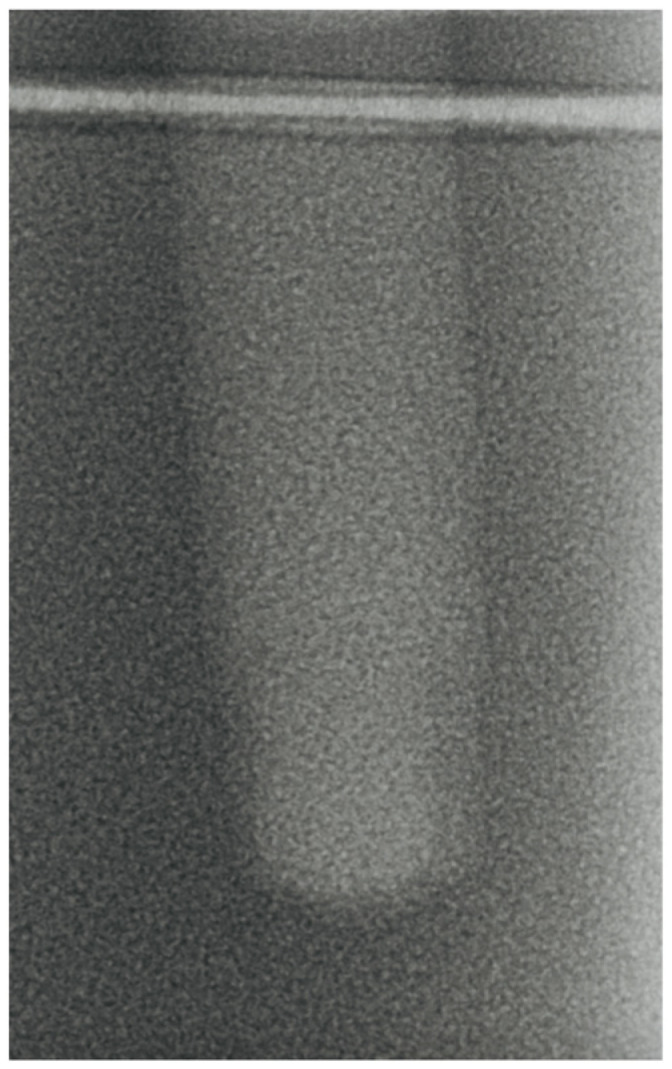
The TD technique (30 PCF).

- Vertical effect of magnetic mallet: The forces produced by the MM are transmitted to the tip of the osteotome in order to achieve the plastic deformation of the bone. These forces can affect the bone in three dimensions: horizontally, vertically, and sagittally. However, the vertical effect of the MM goes beyond the area that is in direct contact with the instrument’s tip. By employing a Micro-CT scan, we accurately measured the extent of condensed bone located apically to the preparation site. The results indicate a direct correlation between the size of the condensed area and the density of the bone. Figure 3 shows an implant prepared site employing the MM technique in a bone density of 30 PCF. The length of our preparation measured 11 mm, while the condensed bone resulting from osteotomy extended 3.7 ± 0.14 mm apically to the implant site preparation. By comparing the MM with the other preparation techniques in the same bone density, we observed that, in the sites prepared using the PES approach, the vertical effect of the preparation was noticeably less than that of the MM. It extended apically to the implant site preparation at 0.36 ± 0.08 mm, as shown in Figure 4. When employing the TD technique, we noticed that the vertical effect of this preparation method was minimal compared to the MM and PES, measuring 0.15 ± 0.04 mm, as shown in Figure 5.

**Figure 6:**
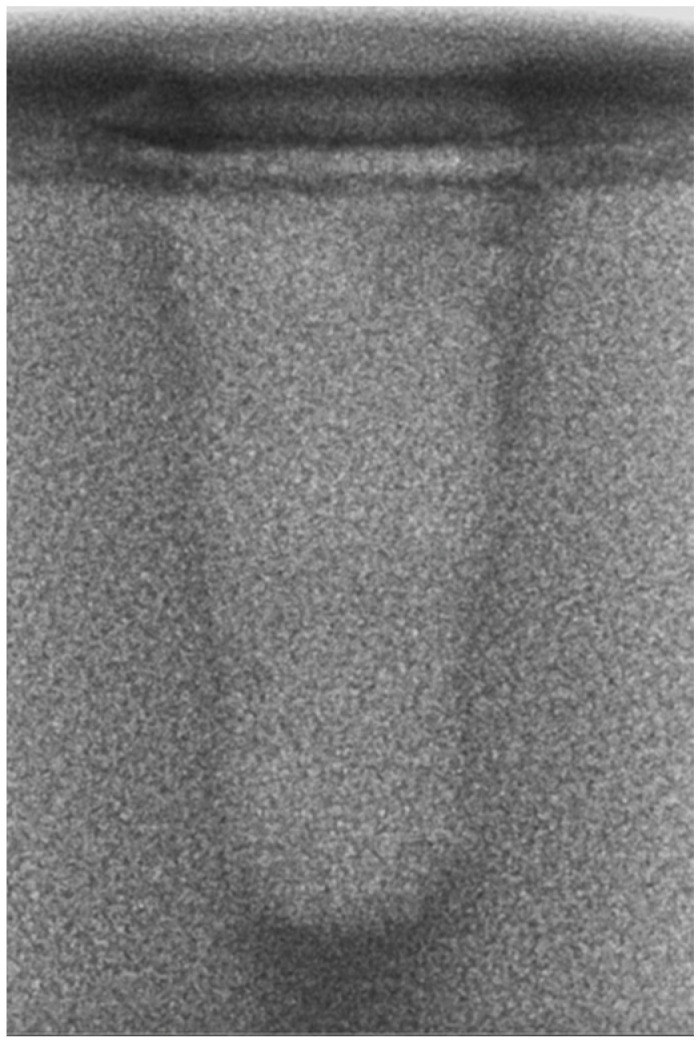
The MM technique (15 PCF).

**Figure 7:**
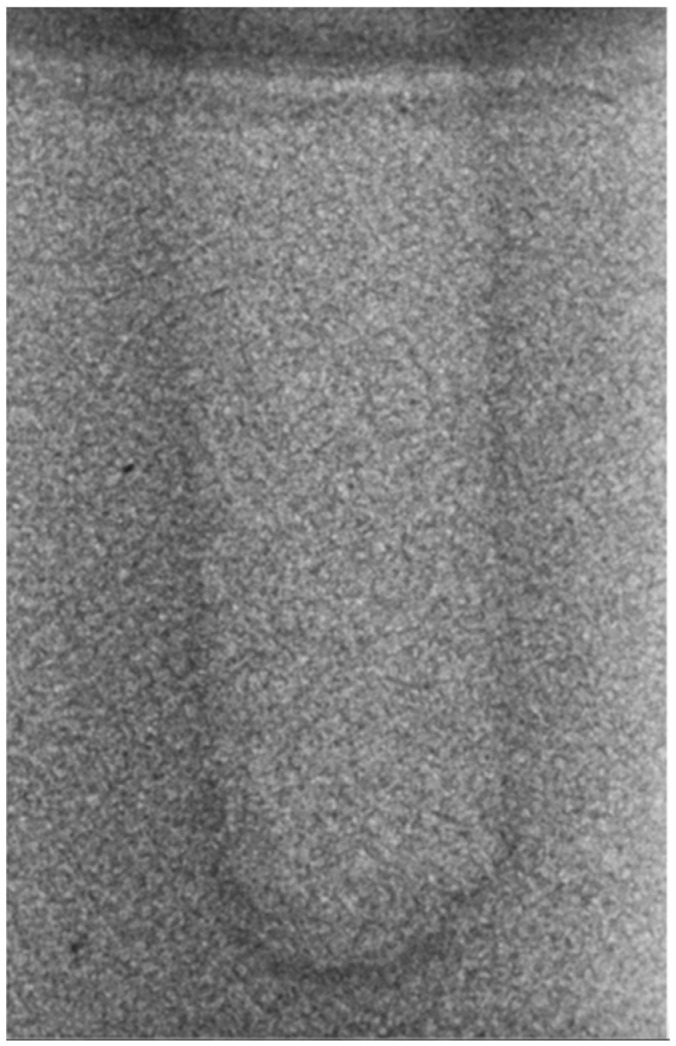
The PES technique (15 PCF).

**Figure 8:**
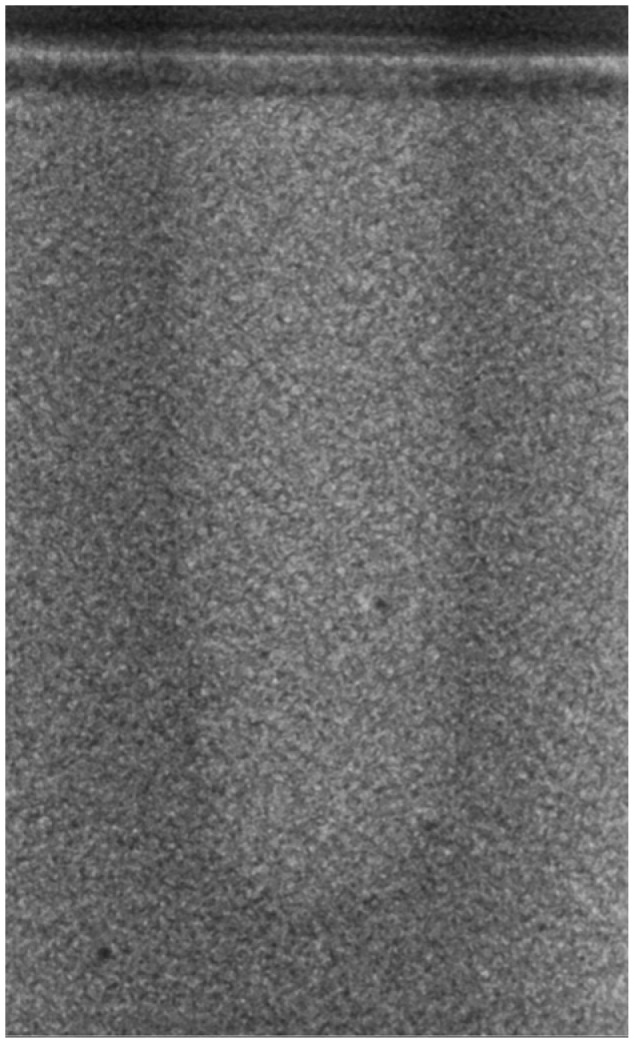
The TD technique (15PCF).

Regarding the bone density of 15 PCF, Figure 6 demonstrates a prepared site utilizing the MM technique. The condensed bone present in this setting exhibited a reduced area in comparison to the density of 30 PCF. The preparation length measured 11 mm, whereas the condensed bone extended 1.4 ± 0.11 mm apically to the implant site preparation. By comparing the MM with the other preparation techniques in the same bone density, we observed that, in the sites prepared using the PES approach, the vertical effect of the preparation was significantly lower than that of the MM. It extended apically to the implant site preparation at 0.27 ± 0.06 mm, as shown in Figure 7. When employing the TD technique, we noticed that the vertical effect of this preparation method was minor in comparison to that of the MM and PES, measuring 0.12 ± 0.03 mm, as shown in Figure 8. The complete results are listed in Table 2.

## 4. Discussion

Implant stability plays a fundamental role in osseointegration. The ratio of cortical to medullary bone, and, consequently, bone quality, has a direct effect on primary stability [34], establishing a direct correlation between cortical thickness and ISQ [35]. Other factors, including implant morphology and surface treatment, as well as operator experience, can have a direct effect on primary stability [36].

Many studies have demonstrated that the surgical procedures employed have a substantial influence on the primary stability of the implant [37,38].

The MM, compared to the TD technique, demonstrates a greater tendency to maintain the structure of the trabecular bone and causes less damage to the cortex and spongy bone. Additionally, it enhances the density of the remaining bone [39,40].

The current study has demonstrated that the ISQ values obtained from the preparations performed with the MM were higher than those achieved with the TD techniques, but lower than those obtained with the PES. This study confirms the clinical and histological findings from previous studies on MM, specifically regarding the capacity of dynamic-magnetic technology to induce bone condensation [41]. Consequently, dynamic-magnetic preparation exhibits a superior mechanical stability compared to the preparation employing TD. Furthermore, the movement of the TD is not constant. The creation of the implant site is a direct outcome of erosion, characterized by an elliptical pattern, leading to areas of bone deficiency [42]. The MM is not subject to this limitation. 

Furthermore, when considering the maximum distance between the 3D-printed screw and the wall of the preparation site (referred to as the micro-gap), the sites prepared with the MM produce superior outcomes compared to those using the PES, but less effective outcomes compared to those using the TD. Regarding the vertical effect of the MM, several authors have demonstrated that using the osteotome technique for implant placement not only improves primary stability but could lead to accelerated bone healing compared with conventional implant placement in trabecular bone [43,44,45]. However, other authors have raised concerns about the potential risk of invading and injuring important anatomical structures like the inferior alveolar nerve and the Schneiderian membrane [46]. Furthermore, they have shown that the condensing approach does not lead to enhanced implant stability [47]. This study does not address the influence of the condensing bone created by the MM on the stability of the implant.

According to Buchter et al. [48], there was no difference in the primary stability of implants placed in porcine bone sites prepared using conventional drills and osteotomes.

In another study comparing conventional drills and osteotomes using dog bone as a model [49], the osteotome group demonstrated a greater primary stability at weeks 0 and 3, with no significant differences at week 8. 

Most of the clinical investigations comparing conventional and osteotome preparations did not find significant differences in ISQ scores [50,51]. In this research, the average ISQ score of the MM was greater than 65, indicating good primary stability in terms of RFA.

When comparing the PES and TD, it has been found that the ultrasonic approach generates around 30,000 micro-vibrations per minute, whereas milling devices produce only a few hundred. The PES enhances instrument control during ostectomy, consequently minimizing the danger of harming the adjacent soft tissues, sinus membranes, nerves, and arteries during surgery [52,53]. The current study clearly demonstrates that the ISQ values obtained from the preparations performed using the PES are higher than those obtained from the preparations performed using the TD and MM. Furthermore, Micro-CT investigations indicate that the ratio of screw to prepared site occupation is greater in the preparations performed with the PES compared to those using the TD and MM. This is associated with a reduction in micro-movements, leading to the improved primary stability of the implants and a higher success rate [54]. This study validates the clinical and histological findings on the excellent primary stability of implants prepared using PES technology, as reported in prior studies on PES [55,56].

As reported by Preti’s study group [57], piezo surgery preparation appears to increase the implant’s stability, as compared to the conventional technique, and promotes the improved healing of the implant site in the early stages, with bone remodeling beginning as early as 56 days after implant insertion.

da Silva Neto et al. [58] showed significantly higher stability in the piezo surgery preparation group in comparison with the conventional drill preparation group at the time of implant placement (77.5 vs. 69.1; *p <* 0.05), after 90 days (77 vs. 70.7; *p <* 0.05), and after 150 days (79.1 vs. 71.7; *p <* 0.05).

It remains a fact that obtaining standardized samples with comparable bone densities in the laboratory setting is extremely challenging. Considering this, rigid polyurethane foam samples covered by a layer of synthetic cortical bone could represent a viable alternative to routinely utilized materials like cadaveric bone samples.

However, synthetic bone can only simulate the mechanical properties of human bone. Another limitation is the correction of artifacts, which is one of the main challenges in the field of X-ray computed tomography at present. In reality, obtaining pictures of the bone–implant interface was not possible, due to the beam-hardening effect caused by the titanium implant.

## 5. Conclusions

Within the limitations of this study, we are able to conclude that the primary stability of the implants is correlated with both the insertion techniques and the bone density. In this study, computed tomography was used to evaluate the macrogeometry of implant site preparations produced by various techniques in polyurethane blocks of two different densities.

This study demonstrates the applicability of the MM in the surgical procedure of implant site preparation, while following the surgical principles outlined by the current literature. These criteria include preserving the integrity of the cortical bone, ensuring sufficient blood circulation, and achieving an aesthetically appropriate result. Therefore, the use of this instrument is a valid alternative to conventional surgical approaches. The concept behind the use of the dynamic MM is to create an optimal implant site preparation and to acquire lateral condensation of the bone while preserving the available cortical bone.

The findings of this study did not show a notable difference between the three preparation techniques in terms of primary stability; however, both the piezoelectric group and the osteo-densification system demonstrated somewhat higher ISQ values than the TD technique group. It can be deduced that, in the case of implant placement with immediate loading, the MM and PES may provide greater opportunities for the early loading of the implants.

Therefore, further clinical studies are necessary to gain a more comprehensive understanding of the biomechanical mechanisms that influence primary stability and bone implant contact in relation to different implant protocols and bone quality.

## Figures and Tables

**Table 1:** ISQ measurement mean and SD in bone density 15 and 30 PCF, with *p*-value < 0.05 for all groups.

**ISQ measurement**	**Bone Density**	**15 PCF** **(Mean ± SD)**			**30 PCF** **(Mean ± SD)**		
	**Groups**	TD	PES	MM	TD	PES	MM
	**Outcomes**	60.6 ± 1.81	72.3 ± 1.63	62.4 ± 1.77	65.8 ± 1.5	75.6 ± 1.73	69.8 ± 1.91

**Table 2:** Micro-CT measurement mean and SD in bone density 15 and 30 PCF, with *p*-value < 0.05 for all groups.

**Micro-CT** **measurement**	**Bone Density**	**15 PCF** **(Mean ± SD)**			**30 PCF** **(Mean ± SD)**		
	Groups	TD	PES	MM	TD	PES	MM
	Occupation ratio (Percent)	90 ± 1.31	89.6 ± 1.22	88.4± 1.17	94 ± 1.88	96 ± 1.75	90.3 ± 2.11
	Maximum Micro-gap (μm)	238 ± 17	318 ± 21	301 ± 20	221 ± 16	299 ± 20	281 ± 19
	Vertical Effect (mm)	0.12 ± 0.03	0.27 ± 0.06	1.4 ± 0.11	0.15 ± 0.04	0.36 ± 0.08	3.7 ± 0.14

## Data Availability

Dataset available on request from the authors.
